# Knockdown of *tgfb1a* partially improves ALS phenotype in a transient zebrafish model

**DOI:** 10.3389/fncel.2024.1384085

**Published:** 2024-04-05

**Authors:** David Gonzalez, Xiomara Cuenca, Miguel L. Allende

**Affiliations:** ^1^Departamento de Ciencias Químicas y Biológicas, Facultad de Ciencias de la Salud, Universidad Bernardo O’Higgins, Santiago, Chile; ^2^Escuela de Terapia Ocupacional, Facultad de Ciencias de la Salud, Universidad Bernardo O’Higgins, Santiago, Chile; ^3^Millennium Institute Center for Genome Regulation, Facultad de Ciencias, Universidad de Chile, Santiago, Chile

**Keywords:** amyotrophic lateral sclerosis, transforming growth factor type β, danio rerio, zebrafish, motor neuron, neurodegenerative disease

## Abstract

Amyotrophic lateral sclerosis (ALS) corresponds to a neurodegenerative disorder marked by the progressive degeneration of both upper and lower motor neurons located in the brain, brainstem, and spinal cord. ALS can be broadly categorized into two main types: sporadic ALS (sALS), which constitutes approximately 90% of all cases, and familial ALS (fALS), which represents the remaining 10% of cases. Transforming growth factor type-β (TGF-β) is a cytokine involved in various cellular processes and pathological contexts, including inflammation and fibrosis. Elevated levels of TGF-β have been observed in the plasma and cerebrospinal fluid (CSF) of both ALS patients and mouse models. In this perspective, we explore the impact of the TGF-β signaling pathway using a transient zebrafish model for ALS. Our findings reveal that the knockdown of *tgfb1a* lead to a partial prevention of motor axon abnormalities and locomotor deficits in a transient ALS zebrafish model at 48 h post-fertilization (hpf). In this context, we delve into the proposed distinct roles of TGF-β in the progression of ALS. Indeed, some evidence suggests a dual role for TGF-β in ALS progression. Initially, it seems to exert a neuroprotective effect in the early stages, but paradoxically, it may contribute to disease progression in later stages. Consequently, we suggest that the TGF-β signaling pathway emerges as an attractive therapeutic target for treating ALS. Nevertheless, further research is crucial to comprehensively understand the nuanced role of TGF-β in the pathological context.

## 1 Introduction

Amyotrophic lateral sclerosis (ALS) corresponds to a neurodegenerative disease affecting upper (UMNs) and lower motor neurons (LMNs) from brain, brainstem and spinal cord. The degeneration of both UMNs and LMNs leads to muscle wasting, fibrosis and paralysis (Pansarasa et al., 2014; Mancuso and Navarro, 2015). ALS patients usually die within 3–5 years from symptoms onset, mainly because of respiratory failures (Taylor et al., 2016). The identification of mutations in the *SOD1* gene in 1993 marked a significant breakthrough in ALS research (Rosen et al., 1993), and 1 year later the first ALS mouse model was generated, carrying the *SOD1* gene with a glycine-to-alanine substitution at position 93 (Gurney et al., 1994). Approximately 90% of ALS cases manifest as the sporadic form (sALS), while the remaining 10% of cases exhibit a genetic component associated and are referred to as familial ALS (fALS) (Riva et al., 2016; Mejzini et al., 2019). As of now, mutations in several genes, such as *SOD1*, TAR DNA-binding protein 43 (*TARDBP*) and *C9ORF72*, have been linked to ALS (Renton et al., 2014; Taylor et al., 2016). To date, only two drugs, riluzole and edaravone, have received approval in certain countries for the treatment of ALS. Riluzole, which acts as an anti-glutamate agent, has demonstrated the ability to improve survival of ALS patients in clinical trials. However, it remains uncertain whether this extension of survival applies uniformly across various stages of the disease (Fang et al., 2018; Andrews et al., 2020; Feldman et al., 2022). Conversely, edaravone, functioning as an antioxidant, has shown the capacity to slow disease progression in clinical trials. Nevertheless, uncertainties persist regarding its applicability to a broader ALS patient population, and questions about its safety and benefits have arisen (Lunetta et al., 2020; Shefner et al., 2020; Feldman et al., 2022; Witzel et al., 2022). Hence, there remains an urgent need to identify new therapeutic targets for the treatment of neurodegenerative diseases, such as ALS.

Transforming growth factor type β (TGF-β) is a widely expressed cytokine that plays pivotal roles in development, homeostasis (Vander Ark et al., 2018) and pathological conditions such as cancer and fibrosis (Peng et al., 2022). In mammals, three isoforms have been identified: TGF-β1, TGF-β2 and TGF-β3, however, the TGF-β superfamily comprises other ligands such as activins, myostatin, Nodal, among others (David and Massagué, 2018). The Smad-dependent TGF-β pathway, also known as canonical pathway, involves the phosphorylation of Smad2/3 complex by the TGF-β receptor I kinase. Afterward, phosphorylated Smad2/3 complex binds to Smad4 and translocates into the nucleus (Massagué, 1998; Leask and Abraham, 2004). In this regard, studies have demonstrated increased levels of TGF-β1 in both the serum and cerebrospinal fluid (CSF) of ALS patients (Houi et al., 2002; Iłz̈ecka et al., 2002) and in an ALS mouse model (Zubiri et al., 2018). Furthermore, increased expression of *Tgfb1* mRNA has been observed in skeletal muscle of ALS mice (Galbiati et al., 2012) and elevated levels of TGF-β cytokine and its canonical downstream mediator, Smad3, have been correlated with muscle fibrosis in these mice (Gonzalez et al., 2017). More recently, the *TGFB1* mRNA has been found to be increased in skeletal muscle of both male and female ALS patients (Meroni et al., 2019). Interestingly, the upregulation of astrocyte-derived TGF-β1 has also been identified in the spinal cord of symptomatic ALS mice, and its pharmacological inhibition has been shown to extend the survival of ALS mice (Endo et al., 2015; Endo and Yamanaka, 2015). A previous study demonstrated that TGF-β1 immunoreactivity is present in the meninges and choroid plexus in the brains of adult rats, as well as in the connective tissue of peripheral nerves and ganglia. However, the authors did not detect expression of TGF-β1 within neurons or glia (Unsicker et al., 1991). Nonetheless, high levels of TGF-β1 mRNA have been identified in several brain areas, including the cerebral cortex, hippocampus, amygdala, hypothalamic paraventricular nucleus, and motor neurons (Vincze et al., 2010). Another study revealed that type I and type II TGF-β receptors are expressed in meningeal fibroblasts following injury. Interestingly, reactive astrocytes did not exhibit strong expression of TGF-β receptors, suggesting that fibroblasts are more likely to respond to TGF-β1 after injury in the central nervous system (Komuta et al., 2010). While there have been some efforts to elucidate the precise role of TGF-β1 signaling in the progression of ALS, several questions remain open.

Zebrafish is emerging as an attractive model for studying neurodegenerative diseases due to its high conservation of genes linked to ALS, genes involved in the development and maintenance of the nervous system, cost-effectiveness, and suitability for high-throughput screening, attributes that distinguish it from other mammalian models (Bandmann and Burton, 2010; Becker and Rinkwitz, 2012). Additionally, the optical transparency of zebrafish embryos is a significant advantage in the context of ALS (Lieschke and Currie, 2007; Babin et al., 2014; Oliveira et al., 2023). This property enables *in vivo* whole-mount microscopy, allowing researchers to visualize motor neurons in real-time. Zebrafish also share structural neuroanatomical and neurophysiological similarities with humans, making them highly relevant for studying ALS. Furthermore, ALS-related locomotor tests can be performed in larvae, such as touch-evoked responses and spontaneous movement assays. These behavioral assays provide valuable insights into motor function, which facilitates the evaluation of potential therapeutic interventions (Babin et al., 2014; Oliveira et al., 2023).

In this perspective, we sought to explore the potential involvement of TGF-β1 in the progression of ALS, using a transient zebrafish model expressing the mutated hSOD1. We also discussed the recent findings regarding the role of TGF-β signaling pathway in ALS progression. Lastly, we offered insights into the future directions and discussed the therapeutic potential of targeting TGF-β1 as a promising avenue for ALS.

## 2 Partial prevention of motor axon abnormalities through *tgfb1a* knockdown in a transient ALS zebrafish model

We employed a transient ALS zebrafish model, which consists in microinjecting the human SOD1 mRNA carrying the G93A mutation (hereafter referred to as hSOD1^G93A^). This well-established model has been extensively used to study ALS in zebrafish (Lemmens et al., 2007; Robinson et al., 2019), since it is valued for its cost-effectiveness and rapid generation of outcomes in zebrafish studies. In order to assess the potential involvement of the TGF-β signaling pathway in the progression of ALS in this zebrafish model, we knocked down the *tgfb1a* by co-microinjecting hSOD1^G93A^ mRNA along with an antisense morpholino (MO) targeting *tgfb1a* mRNA (CAGCACCAAGCAAACCAACCTCATA; GeneTools) at the one cell stage (Supplementary File 1). Briefly, we conducted *in vitro* synthesis of hSOD1^WT/G93A^ mRNAs from plasmids pcDNA3.1(+)SOD1WT (Addgene #26397) and pcDNA3.1(+)SOD1G93A (Addgene #26401) using a mMESSAGE mMACHINE^®^ T7 (Ambion #AM1344) and subsequently purified the mRNAs using the MEGAClear™ kit (Ambion, #AM1908).

**FIGURE 1:**
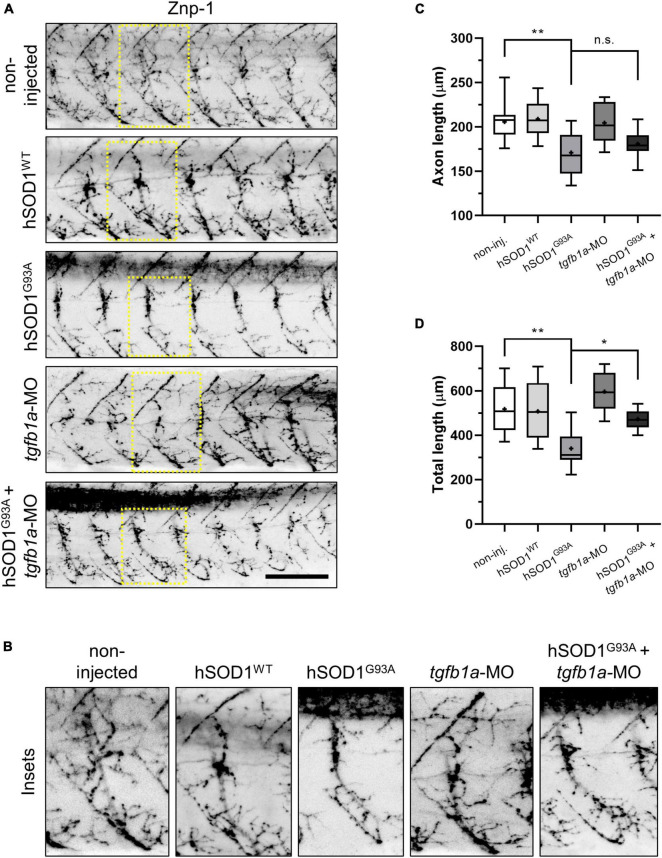
Knockdown of *tgfb1a* partially prevents motor axon abnormalities in hSOD1^G93A^-expressing zebrafish embryos. **(A,B)** Zebrafish embryos at the one-cell stage were subjected to microinjection with hSOD1^WT^ mRNA (*n* = 10), hSOD1^G93A^ mRNA (*n* = 12), *tgfb1a*-MO (*n* = 8), or co-microinjected with hSOD1^G93A^ mRNA and hSOD1^G93A^ mRNA (*n* = 9). Additionally, a non-injected group (*n* = 12) served as a control. At 48 hpf, the embryos were immunostained with Znp-1 antibody, and motor axon morphology was analyzed using ImageJ software with NeuronJ plugin, as previously described (Meijering et al., 2004; Robinson et al., 2019). The graphs depict quantifications of **(C)** axon length and **(D)** total length of motor axons (5–7 motor axons per embryo). Box and whisker plots represent the 25th and 75th percentiles, with medians represented by bisecting lines and means denoted by “+.” Whiskers indicate extreme values. Statistical differences were assessed using one-way ANOVA and Tukey’s multiple comparison test, *p < 0.05, **p < 0.01, n.s.: not significant. Scale bar: 100 μm.

We analyzed the zebrafish motor axons at 48 h post-fertilization (hpf) by immunofluorescence, using a Znp-1 antibody (DSHB Cat# znp-1, RRID:AB_2315626), across the following experimental groups: non-injected, hSOD1^WT^ mRNA, hSOD1^G93A^ mRNA, *tgfb1a*-MO, and hSOD1^G93A^ mRNA + *tgfb1a*-MO. As depicted in the representative images from Figures 1A, B, we observed a significant reduction of both axon length and total length, in hSOD1^G93A^-expressing embryos (171.1 and 340.4 μm), in comparison to non-injected (205.8 and 518 μm) and hSOD1^WT^-expressing embryos (208.9 and 508 μm). While no significant increase in axon length (180.9 μm) was noted in hSOD1^G93A^-expressing embryos co-microinjected with a *tgfb1*-MO (Figure 1C), we found a significant increase in axon total length (473 μm), as illustrated in Figure 1D. These results suggest that the knockdown of *tgfb1a* leads to a partial prevention of motor axon abnormalities in a transient ALS zebrafish model.

## 3 Partial prevention of locomotor deficits through *tgfb1a* knockdown in a transient ALS zebrafish model

**FIGURE 2:**
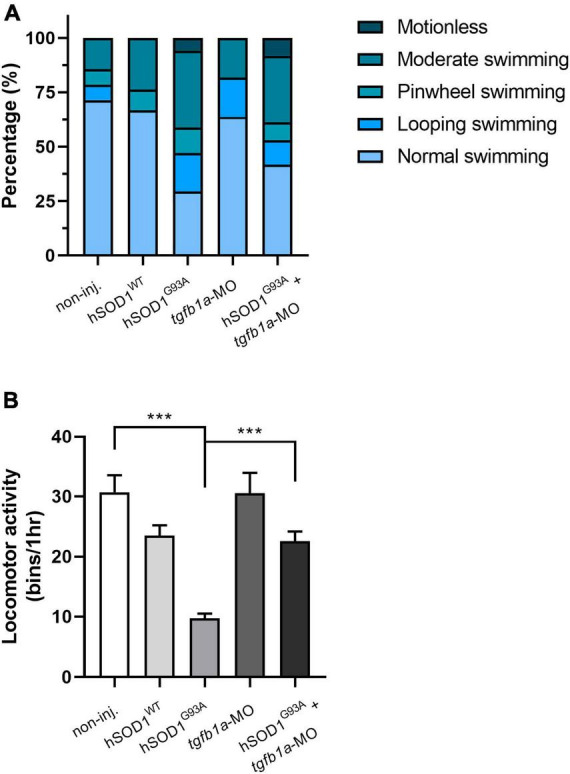
Knockdown of *tgfb1a* partially prevents locomotor deficits in hSOD1^G93A^-expressing zebrafish larvae. At 48 hpf, zebrafish embryos from the following experimental groups: non-injected (*n* = 14), hSOD1^WT^ mRNA (*n* = 14), hSOD1^G93A^ mRNA (*n* = 19), *tgfb1a*-MO (*n* = 11) and hSOD1^G93A^ mRNA + *tgfb1a*-MO (*n* = 11), underwent a **(A)** touch-evoked response assay, involving slight mechanical stimulation with subsequent evaluation and scoring of swimming behavior as normal swimming, looping swimming, pinwheel swimming or motionless. The graph depicts the percentage of each swimming type for the different experimental conditions. **(B)** Spontaneous locomotor activity was assessed in the same experimental groups as in **A**. At 48 hpf, each zebrafish embryo was placed into a single well of a 96-well plate, and their spontaneous locomotor activity was measured over 1 h using the automated Microtracker system, as previously described (Simonetta and Golombek, 2007; Paredes-Zúñiga et al., 2019). The values in **B** correspond to the mean ± SEM. Statistical differences were assessed using one-way ANOVA and Tukey’s multiple comparison test, ***p < 0.001.

Since we found a partial prevention of motor axon abnormalities in *tgfb1a* knocked down hSOD1^G93A^-expressing zebrafish embryos, we wanted to evaluate the locomotor function in these experimental groups. Briefly, we performed a touch-evoked response assay, in which 48 hpf embryos were subjected to gentle mechanical stimulation, and their swimming behavior was subsequently scored. The categorized swimming behavior included normal swimming, looping swimming, pinwheel swimming, or motionless, depending on the embryos’ reactions to the stimulation. We found that the majority of the non-injected and hSOD1^WT^-expressing embryos exhibited normal swimming behavior, accounting for 71 and 66.7%, respectively. In contrast, only 29.4% of hSOD1^G93A^-expressing embryos showed normal swimming behavior. Notably, other types of swimming patterns emerged in this group, including moderate swimming (35.3%), looping swimming (17.6%), pinwheel swimming (11.8%) and motionless (5.9%), as shown in Figure 2A. Importantly, we observed an increase in normal swimming behavior (41.7%) in hSOD1^G93A^-expressing embryos co-microinjected with *tgfb1*-MO compared to hSOD1^G93A^-expressing embryos. Concurrently, we found a reduction in moderate swimming (30.6%), looping swimming (11.1%), pinwheel swimming (8.3%), although 8.3% of the embryos displayed motionless behavior. Zebrafish embryos microinjected with *tgfb1a*-MO did not exhibit significant variations compared to non-injected and hSOD1^WT^-expressing embryos.

Furthermore, we assessed the spontaneous locomotor activity of the aforementioned experimental groups using the Microtracker system (Phylumtech), as previously described (Simonetta and Golombek, 2007; Paredes-Zúñiga et al., 2019). In brief, 48 hpf zebrafish embryos were individually placed into a well of a 96-well plate, and their spontaneous locomotor activity was measured over the course of 1 h. Figure 2B shows that hSOD1^G93A^-expressing embryos excited reduced spontaneous activity (9.72 bins/hr) in comparison to non-injected (30.71 bins/hr) and hSOD1^WT^-expressing embryos (23.5 bins/hr). Interestingly, hSOD1^G93A^-expressing embryos co-microinjected with *tgfb1*-MO showed an increase in spontaneous locomotor activity (22.5 bins/hr) when compared to their hSOD1^G93A^-expressing counterparts. Zebrafish embryos microinjected with *tgfb1a*-MO did not exhibit significant variations in spontaneous locomotor activity compared to non-injected and hSOD1^WT^-expressing embryos. These findings suggest that knockdown of *tgfb1a* results in a partial prevention of locomotor deficits in hSOD1^G93A^-expressing zebrafish embryos.

## 4 Discussion

Our findings show that the knockdown of *tgfb1* partially prevented motor axon abnormalities and partially enhances locomotor function in hSOD1^G93A^-expressing embryos at 48 hpf. In this regard, some evidence exists of the role of TGF-β signaling pathway in ALS disease. As mentioned earlier, elevated levels of TGF-β1 have been detected in serum and CSF of both ALS patients and animal models (Houi et al., 2002; Iłz̈ecka et al., 2002; Peters et al., 2017; Zubiri et al., 2018). More recently, a study showed that R-loops, structures formed of three-stranded nucleic acid, are depleted in ALS patients carrying a mutation in the senataxin (*SETX*) gene, affecting gene expression including the expression of BAMBI, a negative regulator of TGF-β and ultimately, leading to TGF-β signaling pathway activation (Grunseich et al., 2018). Another study has shown reduced levels of *Tgfb1* mRNA in the spinal cord of hSOD1^G93A^ mice at pre-symptomatic stages (Meroni et al., 2019). Thus, given the accumulating evidence regarding the involvement of the TGF-β signaling pathway in ALS disease progression, it has been recently proposed that decreased levels of TGF-β in the early stages of ALS may diminish its neuroprotective activity, increasing the glutamate-induced excitotoxicity. Conversely, elevated TGF-β levels in later stages of ALS are thought to contribute to heightened microglial activation, neuromuscular junction (NMJ) dismantling, and subsequent skeletal muscle atrophy (Galbiati et al., 2020).

While it is challenging to generalize this assertion to every animal model, our observation of improved motor axon morphology in the transient ALS zebrafish model at an embryonic stage (48 hpf) suggests a potential role of TGF-β in this context. It is important to note that our current approach may not distinctly delineate between early and late stages of ALS. Embryonic zebrafish models have certain limitations, especially when modeling adult-onset diseases such as ALS. However, the phenomenon of disease-acceleration observed in zebrafish models is frequently employed as a strategy to enhance tractability at the expense of construct validity (Oliveira et al., 2023). Furthermore, distinguishing between alterations in developmental processes and neurodegeneration presents a challenge (Babin et al., 2014). Thus, using live imaging microscopy in transient and transgenic ALS zebrafish models could offer valuable insights into addressing this question. In this context, zebrafish models provide a convenient and cost-effective experimental model for drug screening, which requires subsequent validation in mammalian models. Further investigations, including those employing transgenic ALS zebrafish models, could provide a more comprehensive understanding of the temporal dynamics of TGF-β involvement in ALS disease progression. A related study has shown that *tgfb1a* gene is expressed in the posterior lateral line (pLL) primordium, a mechanosensory system involved in prey detection and avoidance (Coombs and Van Netten, 2005; Xing et al., 2015). In this study, the knockdown of *tgfb1a* resulted in reduced neuromast number, migration and deposition, indicating that *tgfb1a* gene essential pLL development (Xing et al., 2015). More recently, it has been shown that inhibition of TGF-β signaling perturbs perineurial glia maturation, a cell type that ensheaths axon-Schwann cell bundles and forms the blood-nerve-barrier (Kucenas, 2015; Morris et al., 2017). Interestingly, another study from the same laboratory has shown that TGF-β signaling pathway, through its downstream mediator connective tissue growth factor-a (ctgfa), is involved in forming the bridge between proximal and distal stumps after motor nerve injury in zebrafish embryos (Arena et al., 2022). However, to date, there is no specific evidence demonstrating the role of TGF-β in ALS zebrafish models. In our transient ALS zebrafish model, TGF-β appears to exert a detrimental effect rather than its neuroprotective role, since its knockdown partially prevents the ALS phenotype. TGF-β activation triggers proinflammatory and neurotoxic responses while promoting extracellular matrix (ECM) deposition, which can lead to in neurodegeneration (Peters et al., 2017).

Recently, a study has found that induced pluripotent stem cell (iPSCs)-derived motor neurons from ALS patients carrying mutations in the *C9ORF72* gene show augmented expression of ECM genes and TGF-β targets. However, they did not find the same pattern in iPSCs-derived motor neurons from ALS patients carrying mutations in the *SOD1* gene (Wong and Venkatachalam, 2019). These findings suggest that TGF-β signaling activation in ALS may also be dependent on gene mutations.

The effect of TGF-β can vary depending on the tissue. It is well known that increased levels of TGF-β in skeletal muscle hamper myofiber regeneration and promote deposition of extracellular matrix components (ECM), which leads to a scar formation known as fibrosis in other muscular disorders (Wynn, 2008; Walton et al., 2017; Ismaeel et al., 2019). In this regard, it has been shown that skeletal muscles of symptomatic ALS mice exhibit enhanced canonical TGF-β signaling, which correlates with ECM deposition and induction of fibro/adipogenic progenitors (FAPs) markers, a cell type responsible for secreting ECM (Gonzalez et al., 2017). Moreover, some studies show that TGF-β1-3 expression are increased in muscles of ALS patients (Pradat et al., 2011; Si et al., 2015; Meroni et al., 2019). Thus, it would be interesting to knockdown *tgf1b* in transgenic ALS zebrafish in order to evaluate the ECM deposition within skeletal muscle and how it affects muscle function.

Efforts have been made to assess the therapeutic potential of targeting TGF-β signaling pathway in ALS mice. It has been shown that intraperitoneal injection of recombinant human TGF-β2 improves the locomotor performance in ALS mice; however, it did not prevent the degeneration of motor neurons and disease progression (Day et al., 2005). Conversely, pharmacological inhibition of the TGF-β signaling pathway using the SB-431542 inhibitor has been demonstrated to increase lifespan in ALS mice (Endo et al., 2015). While intriguing, these paradoxical observations reveal the complex roles of TGF-β signaling pathway in the context of ALS. Endo et al. (2015) also showed that astrocytes upregulate TGF-β1 in the lumbar section of the spinal cord from ALS mice and more importantly, the excess of astrocyte-derived TGF-β1 accelerates the progression of the disease. They hypothesize that adverse effects on motor neurons by inhibiting TGF-β may be minimal. However, they propose that modulation of TGF-β signaling in a cell type-specific manner, i.e., inducing TGF-β signaling pathway in motor neuron to promote its neuroprotective effects and, inhibiting TGF-β in astrocytes to reduce neuroinflammation (Endo et al., 2015). On the other hand, the therapeutic potential of other member of the TGF-β superfamily have also been explored in ALS animal models. The inhibition of myostatin, which is a negative regulator of muscle growth, have been shown to reduce muscle atrophy and improve muscle strength in ALS mice and rats. However, despite these beneficial effects on muscle function, myostatin inhibition did not extend the lifespan of ALS rodents (Holzbaur et al., 2006; Morrison et al., 2009). This suggests that while targeting myostatin may ameliorate certain aspects of ALS disease, it may not be sufficient to prolong survival of patients.

In this scenario, it is plausible to consider that inhibition of the TGF-β signaling pathway could be potentially reducing early neuroinflammation and preventing locomotor deficits in our zebrafish model. However, more studies are needed in order to comprehend the precise mechanism by which TGF-β inhibition contributes to the amelioration of the disease. Peters et al. (2017) proposed that sustained upregulation of TGF-β signaling contributes to motor neuron degeneration through various mechanisms. This includes activating a proinflammatory and neurotoxic response, arresting neuronal stem cells, and promoting fibrotic activity (Peters et al., 2017).

Thus, investigating the effects of pharmacological inhibition of the TGF-β signaling pathway across various stages of the disease using a transgenic ALS zebrafish model could shed light onto the involvement of TGF-β in ALS pathogenesis. Such studies could elucidate the temporal dynamics of TGF-β signaling and its impact on ALS disease progression. Understanding how TGF-β signaling varies over time and its specific impact on different stages of ALS disease may reveal opportunities for therapeutic interventions. Lastly, by elucidating these dynamics, researchers will be able to develop more targeted and effective TGF-β-directed therapies for treating ALS patients.

## 5 Concluding remarks and future directions

In this perspective, we showed that the knockdown of *tgfb1* partially enhances the morphology of motor axons and improves locomotor function in hSOD1^G93A^-expressing embryos at 48 hpf. These findings contribute additional evidence supporting the therapeutic potential of modulating TGF-β signaling pathway to ameliorate the progression of ALS. Certainly, further studies are crucial to fully unravel the involvement of TGF-β signaling pathway in ALS. Our current findings raise several questions: (i) What would be the effect of inhibiting TGF-β in later stages of a transgenic ALS zebrafish model? (ii) What are the specific contributions of the TGF-β derived from motor neurons, glia, or skeletal muscle in this zebrafish model? (iii) Is this effect mediated by canonical or non-canonical downstream mediators? The current evidence emphasizes distinct roles of TGF-β depending on the tissue and the stage of the disease (Galbiati et al., 2020), underscoring the need for a more delicate understanding through additional research. Hence, the modulation of the TGF-β signaling pathway is emerging as an appealing therapeutic approach for treating ALS, although more studies are needed to fully comprehend its specific contribution to disease progression.

## Data availability statement

The raw data supporting the conclusions of this article will be made available by the authors, without undue reservation.

## Ethics statement

The animal study was approved by the Animal Use and Care Committee of the University of Chile. The study was conducted in accordance with the local legislation and institutional requirements.

## Author contributions

DG: Conceptualization, Formal analysis, Funding acquisition, Investigation, Methodology, Writing – original draft, Writing – review and editing. XC: Formal analysis, Investigation, Writing – review and editing. MA: Funding acquisition, Resources, Supervision, Writing – review and editing.
