# Supplementary material for: Knockdown of tgfb1a partially improves ALS phenotype in a transient zebrafish model
Source: Front Cell Neurosci. 2024 Apr 5;18:1384085. doi: 10.3389/fncel.2024.1384085 (PMC11032012; doi:10.3389/fncel.2024.1384085)
Supplement: Supplementary file 1 [file Table_1.docx]

**Supplementary Data**

**Materials & Methods**

*Animals*. Zebrafish (*Danio rerio*) *TAB5* were maintained in our facility with a constant photoperiod of 14/10-h ligh-dark at 28°C, with lights turning on at 9 am. Water conditions were kept at pH 7.0 ± 0.3 and conductivity between 600 and 800µS.

*Plasmids and in vitro mRNA transcription*. hSOD1^WT^ and hSOD1^G93A^ mRNAs were synthesized from plasmids pcDNA3.1(+)SOD1WT (Addgene #26397) and pcDNA3.1(+)SOD1G93A (Addgene #26401) using a mMESSAGE mMACHINE^®^ T7 (Ambion #AM1344) and subsequently purified with the MEGAClear^TM^ kit (Ambion, #AM1908).

*Microinjections*. Briefly, 1-2nL of either 250ng/µL hSOD1 WT mRNA, 250ng/µL hSOD1 G93A mRNA, 0.6mM of *tgfb1a*-MO (CAGCACCAAGCAAACCAACCTCATA; GeneTools) were microinjected (or co-microinjected in case of hSOD1 G93A mRNA + *tgfb1*-MO) at one-cell stage, and embryos were maintained in E3 medium at 28°C until 48 hours post-fertilization (hpf).

*Immunofluorescence and confocal microscopy of zebrafish embryos*. At 48hpf, zebrafish embryos were anesthetized with 0.0168% of tricaine and then fixed in 4% PFA for 4 hours at RT. Then, the embryos were permeabilized in 1% Triton X-100 in 1X PBS for 2 hours and blocked in 1% BSA, 1% donkey serum, 0.7% Trition X-100, 1% DMSO in PBS for 1 hour at RT. The embryos were incubated in Znp-1 (DSHB, 1:100) overnight at 4°C. Next day, the embryos were washed in 0.1% Triton X-100 in PBS and incubated in anti-mouse Alexa Fluor 488 (1:500). Acquisition of whole mount immunofluorescences were obtained by using a confocal laser scanning microscope Zeiss LSM710. Quantification of motor axon morphology was performed with ImageJ software using the NeuronJ plugin. Briefly, 12-15 images per embryo were z-stacked (2µm interval) and the axon length was quantified in a semiautomatic fashion, as previously described (Meijering et al., 2004; Robinson et al., 2019).

*Touch-evoked response assay and spontaneous locomotor activity*. At 48hpf, embryos were slightly stimulated in the tail and then scored depending on their swimming behavior as normal swimming, looping swimming, pinwheel swimming or motionless. On the other hand, the spontaneous locomotor activity was measured using the Microtracker system (Phylumtech). Briefly, embryos were placed into a 96-well plate and their spontaneous activity was measured for one hour.

*Statistical analyses*. The statistical significances of the differences among experimental conditions were determined using GraphPad Prism 8 software. In Figure 1, data are presented as box and whisker plots, which represents the 25^th^ and 75^th^ percentile, with medians represented by bisecting lines and means denoted by ‘+’, whereas whiskers indicate extreme values and statistical differences were assessed using one-way ANOVA and Tukey’s multiple comparison test. In Figure 2B, the values correspond to the mean ± SEM and statistical differences were assessed using one-way ANOVA and Tukey’s multiple comparison test.
